# Cooperative TRAIL production mediates IFNα/Smac mimetic-induced cell death in TNFα-resistant solid cancer cells

**DOI:** 10.18632/oncotarget.6915

**Published:** 2016-01-13

**Authors:** Stefanie Roesler, Ines Eckhardt, Sebastian Wolf, Simone Fulda

**Affiliations:** ^1^ Institute for Experimental Cancer Research in Pediatrics, Goethe-University, Frankfurt, Germany; ^2^ German Cancer Consortium (DKTK), Heidelberg, Germany; ^3^ German Cancer Research Center (DKFZ), Heidelberg, Germany

**Keywords:** Smac, apoptosis, cell death, interferon, TRAIL

## Abstract

Smac mimetics antagonize IAP proteins, which are highly expressed in several cancers. Recent reports indicate that Smac mimetics trigger a broad cytokine response and synergize with immune modulators to induce cell death. Here, we identify a differential requirement of TRAIL or TNFα as mediators of IFNα/Smac mimetic-induced cell death depending on the cellular context. Subtoxic concentrations of Smac mimetics cooperate with IFNα to induce cell death in various solid tumor cell lines in a highly synergistic manner as determined by combination index. Mechanistic studies show that IFNα/BV6 cotreatment promotes the formation of a caspase-8-activating complex together with the adaptor protein FADD and RIP1. Assembly of this RIP1/FADD/caspase-8 complex represents a critical event, since RIP1 silencing inhibits IFNα/BV6-induced cell death. Strikingly, pharmacological inhibition of paracrine/autocrine TNFα signaling by the TNFα scavenger Enbrel rescues HT-29 colon carcinoma cells, but not A172 glioblastoma cells from IFNα/BV6-induced cell death. By comparison, A172 cells are significantly protected against IFNα/BV6 treatment by blockage of TRAIL signaling through genetic silencing of TRAIL or its cognate receptor TRAIL receptor 2 (DR5). Despite this differential requirement of TNFα and TRAIL signaling, mRNA and protein expression is increased by IFNα/BV6 cotreatment in both cell lines. Interestingly, A172 cells turn out to be resistant to exogenously added recombinant TNFα even in the presence of BV6, whereas they display a high sensitivity towards TRAIL/BV6. In contrast, BV6 efficiently sensitizes HT-29 cells to TNFα while TRAIL only had limited efficacy. This demonstrates that a differential sensitivity towards TRAIL or TNFα determines the dependency on either death receptor ligand for IFNα/Smac mimetic-induced cell death. Thus, by concomitant stimulation of both death receptor systems IFNα/Smac mimetic combination treatment is an effective strategy to induce cell death in TNFα- or TRAIL-responsive cancers.

## INTRODUCTION

Inhibitor of Apoptosis (IAP) proteins are highly expressed in a variety of human cancers and block programmed cell death by inhibiting caspase activation and by modulating nuclear factor-kappaB (NF-κB) signaling via ubiquitination events [1]. Second mitochondria-derived activator of caspases (Smac) is a mitochondrial intermembrane space protein that promotes apoptosis upon its release into the cytosol by binding to and antagonizing IAP proteins [1].

Small-molecule inhibitors of IAP proteins including Smac mimetics have been designed as anticancer therapeutics to engage programmed cell death in cancer cells [1]. Smac mimetics cause autoubiquitination and proteasomal degradation of IAP proteins that harbor a Really Interesting New Gene (RING) domain, in particular cellular Inhibitor of Apoptosis protein (cIAP) proteins, which leads to NF-κB activation, upregulation of NF-κB target genes such as tumor necrosis factor (TNF)α and TNFα-mediated cell death in the presence of Smac mimetics [2-4]. Also, Smac mimetic-induced IAP depletion promotes the formation of two alternative cytosolic cell death complexes referred to as complexes IIa and IIb. Complex IIa is composed of receptor-interacting protein (RIP) 1, FAS-associated death domain protein (FADD) and caspase-8, leading to activation of caspases and apoptosis [5]. When caspase-8 activity is inhibited, RIP1 interacts with RIP3 to form complex IIb and to initiate necroptosis [5]. Thereby, Smac mimetic-stimulated IAP depletion can engage apoptosis as well as necroptosis.

Since the antitumor activity of Smac mimetic monotherapy proved to be limited in the majority of preclinical cancer models, there has been a continued interest to design rational combinations. To this end, dual immunotherapy approaches using Smac mimetics together with immunostimulatory cytokines may offer a promising option. The type I interferon (IFN)α is an immunostimulatory agent that has been shown to exhibit anticancer activity in solid tumors as well as in hematological malignancies [6]. For clinical application, modified IFN products such as pegylated IFNα2 with extended *in vivo* half-lives that constantly increase IFNα levels over prolonged periods of times have been developed [6, 7]. IFNα is considered to exert its anticancer effects both directly via its effects on cancer cells and indirectly via activation of immune cells [8]. Binding of IFNα to the IFNα receptor on the plasma membrane of cancer cells engages signal transduction pathways that lead to inhibition of cell proliferation and/or induction of apoptosis [8]. We recently found that the small-molecule Smac mimetic BV6 together with recombinant IFNα synergistically induces apoptosis in acute myeloid leukemia (AML) cells without increased toxicity against normal peripheral blood lymphocytes [9]. In the present study, we investigated the anticancer activity of this combinatory approach beyond AML in a variety of solid tumors and explored the underlying molecular mechanisms of action.

## RESULTS

### IFNα and BV6 synergistically induce cell death in various cancer cell lines

**Figure 1:**
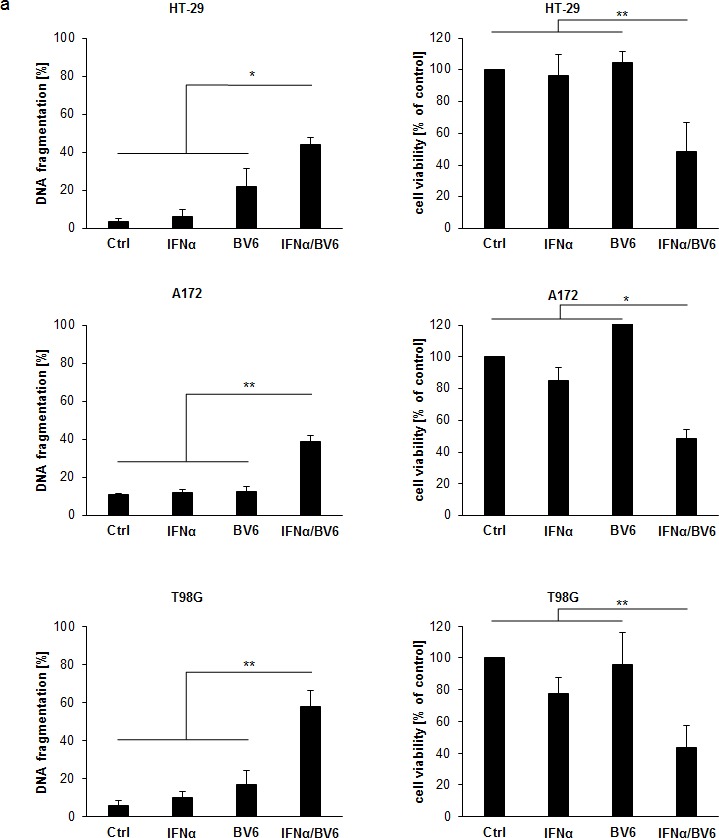

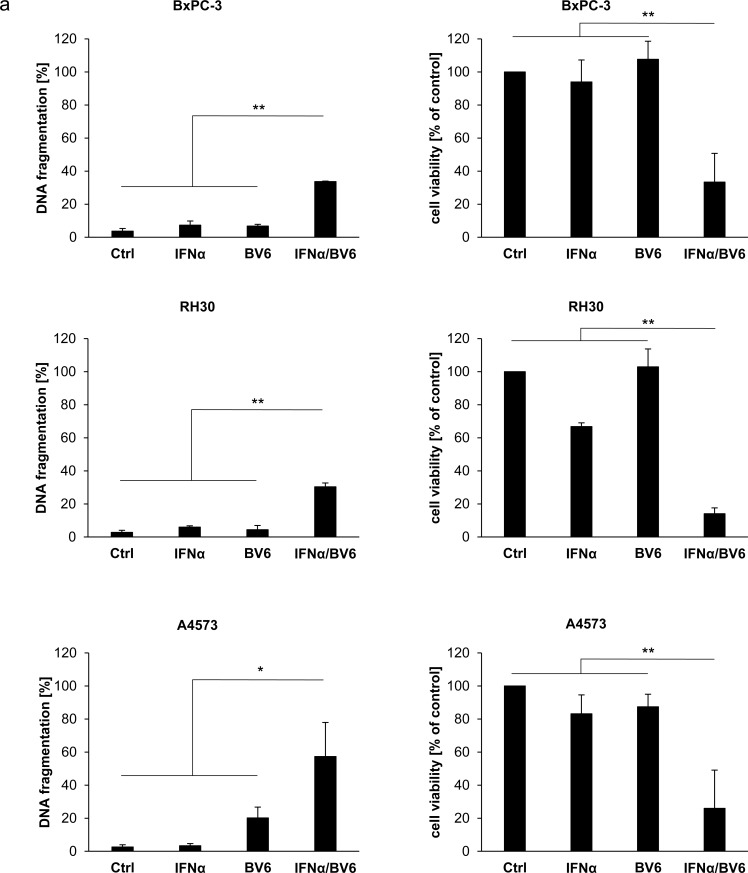

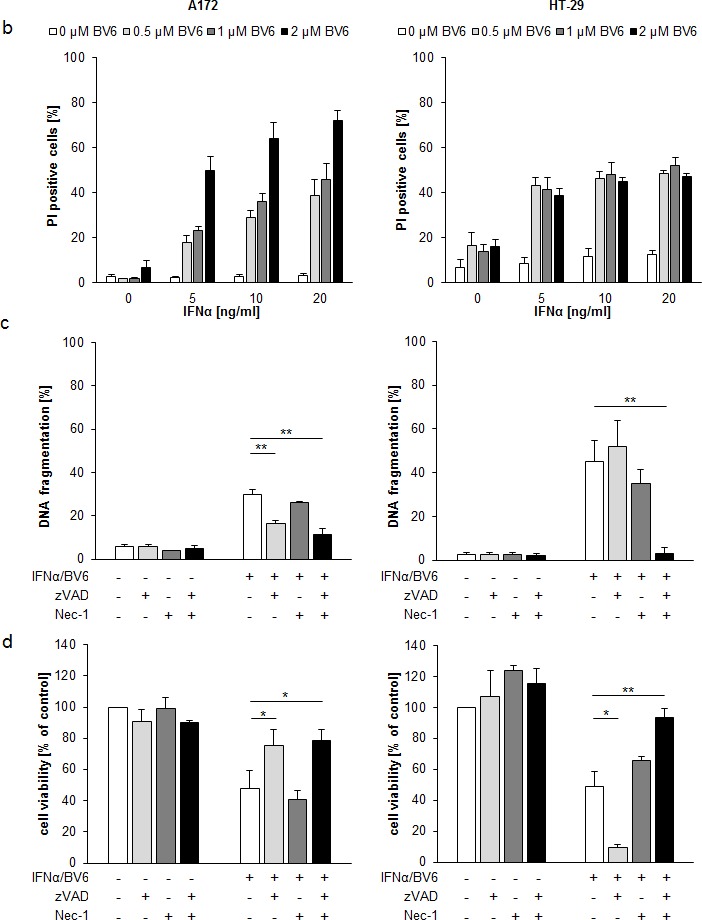
IFNα and BV6 synergistically induce cell death in various cancer cell lines **A.** Cells were treated with IFNα (HT-29, BxPC-3, A4573: 10 ng/ml; A172, T98G, RH30: 5 ng/ml) and/or BV6 (HT-29, A172: 1 μM; T-98G: 7 μM; BxPC-3: 2 μM; RH30: 500 nM; A4573: 4 μM) for 72 hours (except BxPc-3 were treated for 24 hours). Cell death was determined by analysis of DNA fragmentation of PI-stained nuclei using flow cytometry and mean + SD of three independent experiments performed in duplicate are shown (left panels). Cell viability was determined by MTT assay and data are shown as percentage of untreated control cells with mean + SD of three independent experiments performed in triplicate (right panel). **B.** A172 and HT-29 cells were treated for 72 hours with indicated concentrations of IFNα and/or BV6. Cell death was determined by PI staining using flow cytometry and mean + SD of three independent experiments performed in duplicate are shown. **C.**, **D.** Cells were treated for 72 hours with IFNα (A172: 5 ng/ml, HT-29: 10 ng/ml) and 1 μM BV6 in the presence or absence of 20 μM zVAD.fmk and/or 30 μM Nec-1 without renewal of inhibitors. Cell death was determined by analysis of DNA fragmentation of PI-stained nuclei using flow cytometry and mean + SD of three independent experiments performed in duplicate are shown **C.**. Cell viability was determined by MTT assay and data are shown as percentage of untreated control cells with mean + SD of three independent experiments performed in triplicate **D.**. *P < 0.05; **P < 0.01.

Initially, we evaluated the dual immunotherapy approach employing Smac mimetics and recombinant IFNα in a panel of solid cancer cell lines. IFNα and BV6 cooperated to induce apoptosis (determined by DNA fragmentation as a typical marker of apoptotic cell death) and to reduce cell viability (measured by MTT assay) in several cell lines from different cancer entities including HT-29 colon carcinoma, A172 and T98G glioblastoma, BxPC-3 pancreatic carcinoma, RH30 rhabdomyosarcoma and A4573 Ewing sarcoma cells (Figure 1A). We then selected A172 glioblastoma and HT-29 colon carcinoma cells for subsequent studies. Dose-response studies showed that IFNα and BV6 triggered apoptosis and reduced cell viability in a synergistic manner as determined by calculation of combination index (CI) (Suppl. Fig.1; Suppl. Table 1). Synergistic tumoricidal activity of IFNα and BV6 was confirmed using propidium iodide (PI) staining to determine plasma membrane permeabilization as another cell death assay (Figure 1B). To test the general relevance of our findings we extended these experiments to other Smac mimetics. Similarly, monovalent (LCL161, CUDC427) and bivalent (Birinapant) Smac mimetics acted in concert with IFNα to reduce cell viability (Suppl. Figure 2). Together, these data demonstrate that IFNα and Smac mimetics cooperate to induce cell death across different cancer entities.

To determine whether caspase activity is required for IFNα/BV6-induced cell death, we used the pharmacological pan-caspase inhibitor zVAD.fmk. Addition of zVAD.fmk significantly decreased IFNα/BV6-induced DNA fragmentation and increased cell viability in A172 cells (Figure 1C, 1D; left panels). In contrast, IFNα/BV6 in combination with zVAD.fmk even significantly reduced cell viability in HT-29 cells when compared to IFNα/BV6 treatment alone, while we observed no significant increase in DNA fragmentation (Figure 1C, 1D; right panels). These results indicate that A172 cells undergo caspase-dependent apoptosis in response to IFNα/BV6 cotreatment, while HT-29 cells can die via a caspase-independent, non-apoptotic mechanism upon caspase inhibition that is not associated with typical apoptotic markers such as DNA fragmentation.

Therefore, we next investigated whether IFNα/BV6 cotreatment induces necroptosis as an alternative mode of cell death in caspase-inhibited HT-29 cells. To this end, we tested the effects of the RIP1 kinase inhibitor Necrostatin-1 (Nec-1) in the presence and absence of zVAD.fmk. Addition of Nec-1 significantly rescued caspase-inhibited HT-29 cells from IFNα/BV6-mediated loss of cell viability and cell death, while Nec-1 exerted no significant effects on IFNα/BV6-induced loss of cell viability and cell death in HT-29 cells without caspase inhibition (Figure 1C, 1D; right panels) or in A172 cells in the presence or absence of zVAD.fmk (Figure 1C, 1D; left panels). This indicates that IFNα/BV6 cotreatment induces caspase-dependent apoptosis in A172 cells, while HT-29 cells undergo RIP1-dependent necroptosis when caspase activity is impaired.

### IFNα/BV6 cotreatment triggers IAP depletion, complex II formation and caspase activation

**Figure 2:**
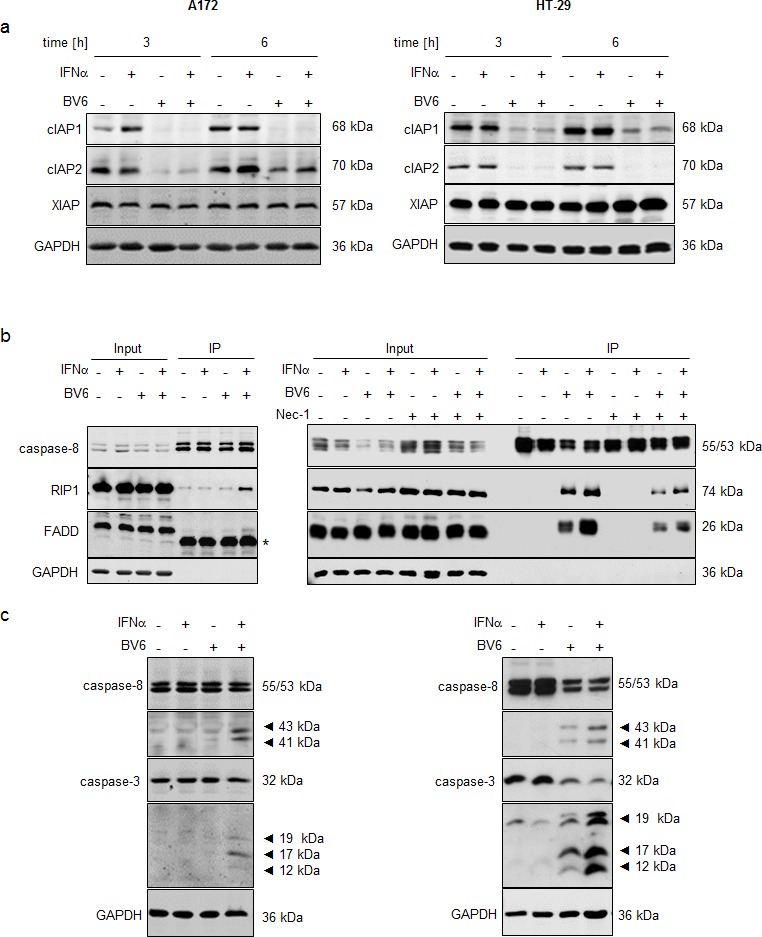
IFNα/BV6 cotreatment induces IAP depletion, complex II formation and caspase activation **A.** Cells were treated for 3 and 6 hours with IFNα (A172: 5 ng/ml, HT-29: 10 ng/ml) and/or 1 μM BV6. Protein levels of cIAP1/2 and XIAP were assessed by Western blotting, GAPDH was used as loading control. **B.** Cells were treated for 12 hours with IFNα (A172: 5 ng/ml, HT-29: 10 ng/ml) and/or 1 μM BV6 in the presence of 20 μM zVAD.fmk and/or 30 μM Nec-1 (HT-29 only). Caspase-8 was immunoprecipitated using an anti-caspase-8 antibody and detection of RIP1, FADD and caspase-8 was done by Western blotting, GAPDH served as loading control. Asterisk indicates IgG light chain. **C.** Cells were treated for 24 hours (A172) and 18 hours (HT-29) with IFNα (A172: 5 ng/ml, HT-29: 10 ng/ml) and/or 1 μM BV6. Cleavage of caspase-8 and 3 was assessed by Western blotting, active cleavage products are indicated by arrows. GAPDH was used as loading control.

Since depletion of IAP proteins is a hallmark of Smac mimetic-induced cell death [2], we next assessed expression levels of cIAP1, cIAP2 and x-linked Inhibitor of Apoptosis Protein (XIAP). Treatment with BV6 alone and in combination with IFN caused rapid downregulation of cIAP1 and cIAP2 already after 3 hours (Figure 2A) and a slight reduction of XIAP protein levels after 12 to 18 hours (Suppl. Figure 3). At 6 hours we noted re-expression of cIAP2 in A172 cells, which was initially degraded at 3 hours (Figure 2A). This rebound of cIAP2 expression upon prolonged exposure to Smac mimetic has previously been linked to Smac mimetic-stimulated activation of NF-κB [10] and the requirement of cIAP1 for Smac mimetic-triggered degradation of cIAP2.

Since depletion of cIAP proteins favors formation of a cytosolic complex composed of RIP1, FADD and caspase-8, we immunoprecipitated caspase-8 and analyzed its interaction with FADD and RIP1. We used zVAD.fmk to limit caspase-8 activity and to stabilize the complex. To avoid a switch to necroptosis in the prototypic necroptosis prone cell line HT-29, we additionally combined zVAD.fmk with Nec-1 to differentiate complex formation under apoptotic and necroptotic conditions. Interestingly, IFNα and BV6 cooperated to stimulate interaction of RIP1, FADD and caspase-8 in both A172 and HT-29 cells (Figure 2B). HT-29 cells also displayed assembly of RIP1, FADD and caspase-8 upon treatment with BV6 alone (Figure 2B, right panel), consistent with the higher sensitivity of HT-29 cells compared to A172 cells towards BV6 (Figure 1B). The assembly of RIP1, FADD and caspase-8 in HT-29 cells was decreased in the presence of Nec-1 (Figure 2B, right panel), indicating an involvement of RIP1 kinase activity. Furthermore, IFNα and BV6 cooperated to induce cleavage of caspase-8 into p43/p41 fragments and cleavage of caspase-3 into p19/p17/p12 fragments (Figure 2C). Together, this set of experiments demonstrates that IFNα/BV6 cotreatment triggers IAP depletion, complex II formation and caspase activation.

### IFNα/BV6-induced cell death depends on RIP1

**Figure 3:**
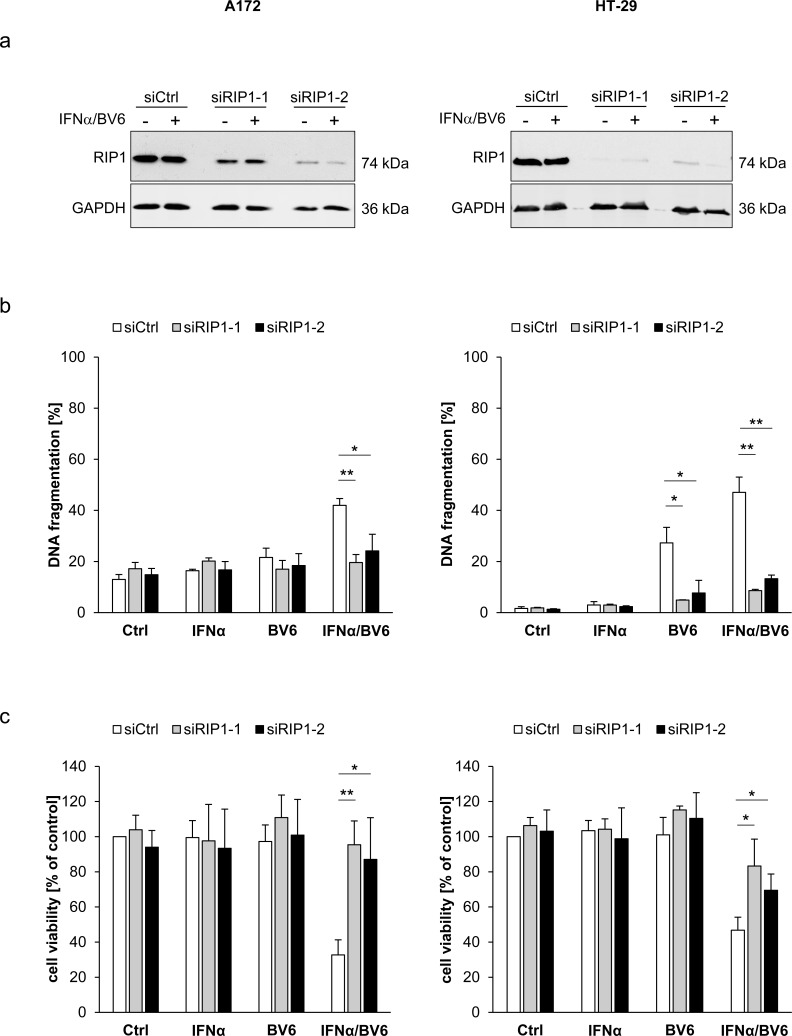

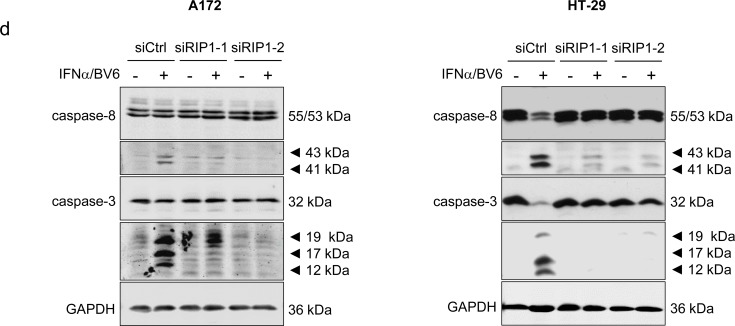
IFNα/BV6-induced cell death depends on RIP1 Cells were transiently transfected with 5 nM siRNA targeting RIP1 (siRIP1-1, siRIP1-2) or control siRNA. **A.** Cells were treated with IFNα (A172: 5 ng/ml, HT-29: 10 ng/ml) and 1 μM BV6. Protein expression of RIP1 was analyzed by Western blotting, GAPDH was used as loading control. **B.** Cells were treated for 72 hours with IFNα (A172: 5 ng/ml, HT-29: 10 ng/ml) and/or 1 μM BV6. Cell death was determined by analysis of DNA fragmentation of PI-stained nuclei using flow cytometry. Mean + SD of three independent experiments performed in duplicate are shown; *P < 0.05; **P < 0.01. **C.** Cell viability was determined by MTT assay. Data are shown as percentage of untreated control cells with mean + SD of three independent experiments performed in triplicate; *P < 0.05; **P < 0.01. **D.** Cells were treated for 24 hours with IFNα (A172: 5 ng/ml, HT-29: 10 ng/ml) and 1 μM BV6. Cleavage of caspase-8 and 3 was assessed by Western blotting, GAPDH was used as loading control.

Since scaffolding functions of RIP1 in addition to its kinase activity have been reported to be indispensable for the induction of cell death [5], we genetically silenced RIP1 using two independent siRNA sequences directed against RIP1. Knockdown efficacy of RIP1 was confirmed by Western blotting (Figure 3A). Of note, silencing of RIP1 significantly protected A172 and HT-29 cells against IFNα/BV6-induced DNA fragmentation as well as loss of cell viability (Figure 3B, 3C). Also, RIP1 knockdown reduced IFNα/BV6-mediated cleavage of caspase-8 and -3 into active cleavage fragments (Figure 3D). This set of experiments demonstrates that RIP1 is required for IFNα/BV6-induced cell death.

### Differential requirement of TNFα for IFNα/BV6-induced cell death

**Figure 4:**
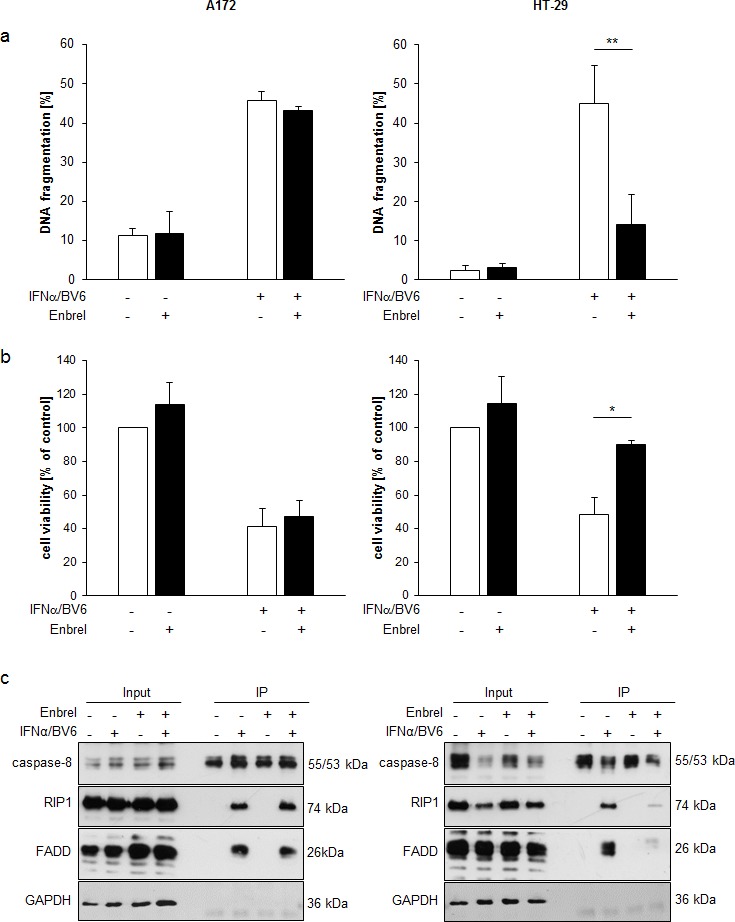
Differential requirement of TNFα for IFNα/BV6-induced cell death **A.**, **B.** Cells were treated for 72 hours with IFNα (A172: 5 ng/ml, HT-29: 10 ng/ml) and 1 μM BV6 in the presence or absence of 50 μg/ml Enbrel. Cell death was determined by analysis of DNA fragmentation of PI-stained nuclei using flow cytometry and mean + SD of three independent experiments performed in duplicate are shown **A.**. Cell viability was determined by MTT assay and data are shown as percentage of untreated control cells with mean + SD of three independent experiments performed in triplicate **B.**; *P < 0.05; **P < 0.01. **C.** Cells were treated for 12 hours with IFNα (A172: 5 ng/ml, HT-29: 10 ng/ml) and 1 μM BV6 and/or 50 μg/ml Enbrel in the presence of 20 μM zVAD.fmk. Caspase-8 was immunoprecipitated using an anti-caspase-8 antibody and detection of RIP1, FADD and caspase-8 was done by Western blotting, GAPDH was used as loading control.

To test whether TNFα is involved in mediating cell death upon IFNα/BV6 combinatorial therapy, we used the TNFα-blocking antibody Enbrel. Indeed, administration of Enbrel significantly decreased IFNα/BV6-induced DNA fragmentation and loss of cell viability in HT-29 cells (Figure 4A, 4B; right panels). In contrast, Enbrel failed to rescue A172 cells from IFNα/BV6 cotreatment (Figure 4A, 4B; left panels). Consistently, Enbrel suppressed IFNα/BV6-stimulated formation of the RIP1/FADD/caspase-8 complex in HT-29 cells but not in A172 cells (Figure 4C). These findings indicate a differential requirement of TNFα for IFNα/BV6-induced cell death.

### IFNα and BV6 cooperate to increase TNFα and TRAIL production

**Figure 5:**
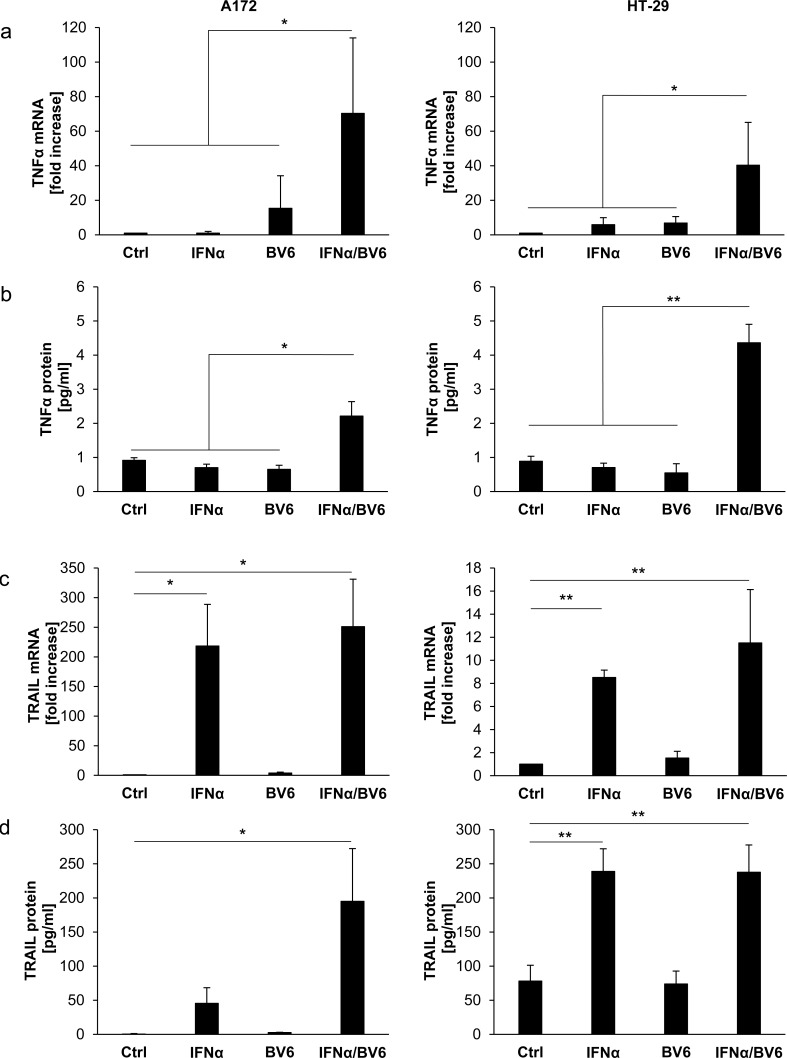
IFNα and BV6 cooperate to increase TNFα and TRAIL production **A.** Cells were treated for 12 hours with IFNα (A172: 5 ng/ml, HT-29: 10 ng/ml) and/or 1 μM BV6. TNFα mRNA levels were determined by qRT-PCR and are shown as fold increase with mean + SD of four independent experiments performed in duplicate; 28S rRNA was used as loading control, *P < 0.05; **P < 0.01. **B.** Cells were treated for 14 hours with IFNα (A172: 5 ng/ml, HT-29: 10 ng/ml) and/or 1 μM BV6. TNFα protein levels in cell culture supernatants were determined by ELISA and are shown as protein concentration with mean + SD of four independent experiments performed in duplicate; *P < 0.05; **P < 0.01. **C.** Cells were treated for 12 hours with IFNα (A172: 5 ng/ml, HT-29: 10 ng/ml) and/or 1 μM BV6. TRAIL mRNA levels were determined by qRT-PCR and are shown as fold increase with mean + SD of three (A172) or four (HT-29) independent experiments performed in duplicate; 28S rRNA was used as loading control, *P < 0.05; **P < 0.01. **D.** Cells were treated for 16 hours with IFNα (A172: 5 ng/ml, HT-29: 10 ng/ml) and/or 1 μM BV6. TRAIL protein levels from cell lysates were determined by ELISA and are shown as protein concentration with mean + SD of four independent experiments performed in duplicate; *P < 0.05; **P < 0.01.

To investigate whether this differential requirement of TNFα for IFNα/BV6-induced cell death is due to differences in TNFα production, we determined TNFα mRNA levels and secretion of TNF protein in response to IFNα/BV6 cotreatment. Interestingly, cotreatment with IFNα/BV6 significantly increased TNFα mRNA expression as well as secretion of TNFα into the supernatant in both A172 and HT-29 cells (Figure 5A, 5B). Since TNFα turned out to be dispensable for IFNα/BV6-induced cell death in A172 cells, we also assessed production of Tumor-Necrosis-Factor-related apoptosis-inducing ligand (TRAIL) as another death receptor ligand besides TNFα in response to IFNα/BV6 cotreatment. Treatment with IFNα alone and in combination with BV6 upregulated TRAIL mRNA levels in both HT-29 and A172 cells (Figure 5C). Also, IFNα and BV6 cooperated to enhance expression of TRAIL protein in both cell lines (Figure 5D). Thus, combined IFNα/BV6 immunotherapy stimulates production of TNFα and TRAIL in both A172 and HT-29 cells.

### IFNα/BV6-induced cell death depends on TRAIL signaling in A172 cells

**Figure 6:**
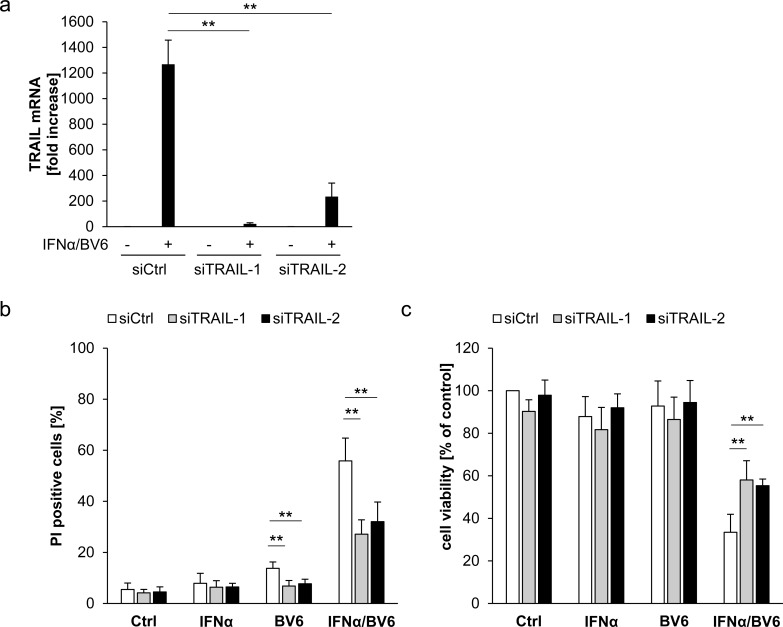

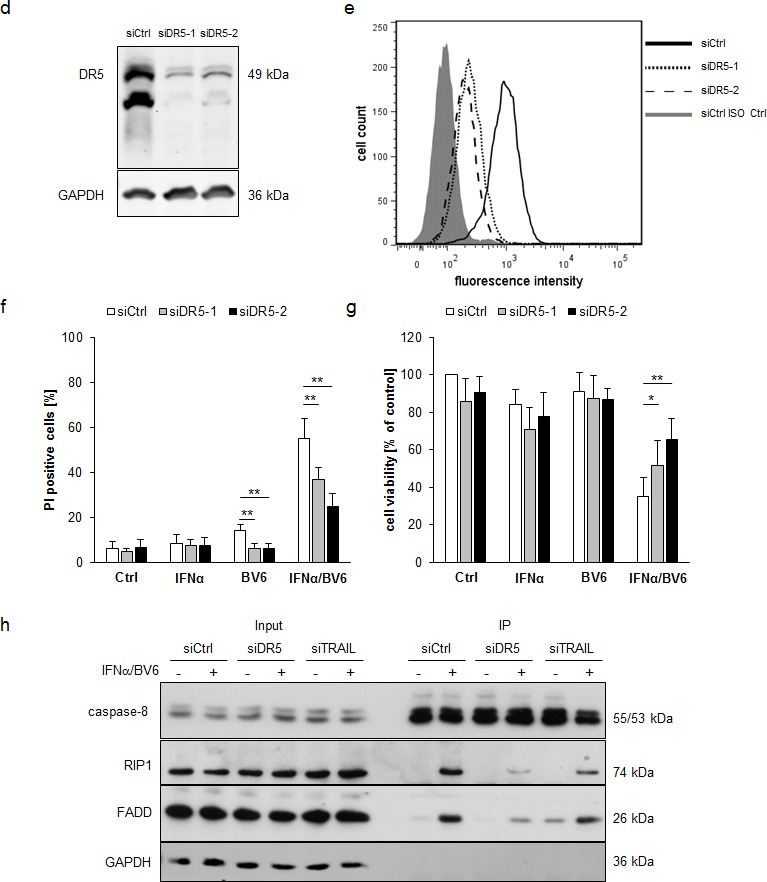
IFNα/BV6-induced cell death depends on TRAIL signaling in A172 cells **A.**-**C.** A172 cells were transiently transfected with 5 nM siRNA targeting TRAIL (siTRAIL1, siTRAIL2) or control siRNA. TRAIL mRNA levels were analyzed by qRT-PCR and are shown as fold increase with mean + SD of three (A172) or five (HT-29) independent experiments performed in duplicate, 28S rRNA was used as loading control **A.**. Cell death was determined after treatment for 72 hours with 5 ng/ml IFNα and/or 1 μM BV6 by PI staining using flow cytometry, mean + SD of five independent experiments performed in duplicate are shown; *, *P* < 0.05; **P < 0.01 **B.**. Cell viability was determined by MTT assay and data are shown as percentage of untreated control cells with mean + SD of five independent experiments performed in triplicate; *P < 0.05; **P < 0.01 **C.**.**D.**-**G.** A172 cells were transiently transfected with 5 nM siRNA targeting DR5 (siDR5-1, siDR5-2) or control siRNA. Protein expression of DR5 was analyzed by Western blot analysis, GAPDH was used as loading control **D.**, and by immunostaining of DR5 surface expression using flow cytometry showing the isotype control (ISO Ctrl) in grey **E.**. Cell death was determined after treatment for 72 hours with 5 ng/ml IFNα and/or 1 μM BV6 by PI staining using flow cytometry and mean + SD of three independent experiments performed in duplicate are shown (F). Cell viability was determined by MTT assay and data are shown as percentage of untreated control cells with mean + SD of three independent experiments performed in triplicate; *P < 0.05; **P < 0.01 (G). **H.** A172 cells were transiently transfected with 5 nM siRNA targeting DR5 (siDR5-2), TRAIL (siTRAIL-1) or control siRNA and treated with 5 ng/ml IFNα and 1 μM BV6 for 18 hours. Caspase-8 was immunoprecipitated using an anti-caspase-8 antibody and detection of RIP1, FADD and caspase-8 was done by Western blotting, GAPDH served as loading control.

To investigate whether TRAIL is required as a mediator of IFNα/BV6-induced cell death in A172 cells, which die in a TNFα-independent manner (Figure 4A-4C), we silenced TRAIL by siRNA oligonucleotides (Figure 6A). Interestingly, knockdown of TRAIL significantly reduced IFNα/BV6-induced cell death as well as loss of cell viability in A172 cells (Figure 6B, 6C). We also used an alternative strategy to block the TRAIL system by silencing DR5, since DR4 is not expressed in A172 cells [11]. Similarly, knockdown of DR5 significantly protected A172 cells against IFNα/BV6-imposed cell death and loss of cell viability (Figure 6D-6G).

Since we found that IFNα/BV6-stimulated assembly of the RIP1/FADD/caspase-8 complex occurs in a TNFα-independent manner in A172 cells (Figure 4C), we then explored whether signaling via TRAIL mediates formation of this cell death complex upon IFNα/BV6 cotreatment. Indeed, knockdown of either DR5 or TRAIL reduced recruitment of RIP1, FADD and caspase-8 compared to control cells (Figure 6H). Together, this set of experiments shows that TRAIL is required for IFNα/BV6-induced formation of the RIP1/FADD/caspase-8 complex and cell death in A172 cells.

### Differential sensitivity to TRAIL and TNFα is responsible for the differential requirement of TRAIL and TNFα for IFNα/BV6-induced cell death

**Figure 7:**
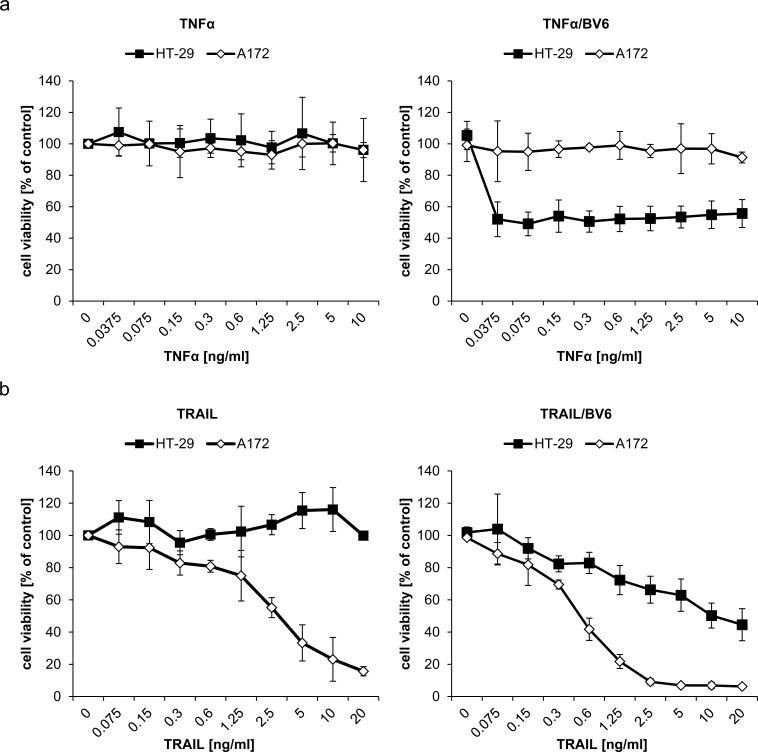
Differential sensitivity to TNFα and TRAIL is responsible for the differential requirement of TNFα and TRAIL for IFNα/BV6-induced cell death A172 and HT-29 cells were treated for 72 hours with indicated concentrations of TNFα **A.** or TRAIL **B.** with (right panel) or without (left panel) 1 μM BV6. Cell viability was determined by MTT assay. Data are shown as percentage of untreated control cells with mean + SD of three independent experiments performed in triplicate; *P < 0.05; **P < 0.01.

Since A172 and HT-29 cells display a differential requirement of TRAIL and TNFα signaling for IFNα/BV6-induced cell death, although they produce comparable amounts of TRAIL and TNFα upon IFNα/BV6 cotreatment (Figure 5B, 5D) and express TNFR1 and DR5 (suppl. Figure 4), we hypothesized that A172 and HT-29 cells are differentially sensitive to these death receptor ligands. To test this hypothesis we exogenously added TNFα or TRAIL at increasing concentrations in the presence and absence of BV6. Strikingly, A172 cells were refractory to treatment with TNFα alone and in the presence of BV6 (Figure 7A). In contrast, addition of BV6 rendered HT-29 cells sensitive to TNFα-induced loss of cell viability even at low concentrations of TNFα (Figure 7A). TRAIL triggered loss of cell viability in A172 cells in a dose-dependent manner, whereas HT-29 cells were resistant even to the highest dose of TRAIL that was used (Figure 7B, left panel). Addition of BV6 further enhanced the TRAIL-induced loss of cell viability in A172 cells (Figure 7B, right panel). While BV6 also enhanced the sensitivity of HT-29 cells towards TRAIL, HT-29 cells remained substantially less responsive towards BV6/TRAIL compared to A172 cells (Figure 7B) with more than 1 log difference in IC50 values (suppl. Figure 5; IC50 A172 cells: 0.49 ng/ml TRAIL, IC50 A HT-29 cells: 9.54 ng/ml TRAIL). Together, these findings show that A172 cells are sensitive to TRAIL but refractory to TNFα, whereas HT-29 cells are susceptible to TNFα in the presence of Smac mimetics.

## DISCUSSION

In the present study, we show that dual immunotherapy employing Smac mimetics and recombinant IFNα represents a promising approach to reactivate cell death in various solid cancers. IFNα and Smac mimetics at suboptimal concentrations act together to trigger cell death in a synergistic manner as confirmed by calculation of CI.

The novelty of our study resides in particular in the following two points. First, we demonstrate that IFNα/Smac mimetic combination treatment is an effective strategy to induce cell death in TNFα- or TRAIL-responsive cancers by concomitant stimulation of both death receptor systems. We identify a differential requirement of TRAIL or TNFα as mediators of IFNα/Smac mimetic-induced cell death depending on the cellular context. We show that the sensitivity to TRAIL and TNFα determines whether a cell line depends on TRAIL or TNFα signaling to mediate IFNα/BV6-induced cell death irrespective of increased production of these death ligands in response to IFNα/BV6 combination therapy. We characterize two prototypic models that require either TRAIL or TNFα for IFNα/BV6-induced cell death. In A172 glioblastoma cells, TRAIL turned out to be the critical mediator of IFNα/BV6-induced cell death, since genetic silencing of TRAIL or DR5 protects cells from cell death upon IFNα/BV6 cotreatment. By comparison, TNFα is a crucial mediator of IFNα/BV6-induced cell death in HT-29 colon carcinoma cells, since pharmacological blockage of TNFα rescues cell death by IFNα/BV6. In both cell line models, the addition of suboptimal concentrations of IFNα to subtoxic doses of Smac mimetics results in the cooperative production of TRAIL and TNFα. Interestingly, the cell's sensitivity to TRAIL or TNFα rather than the production of these death receptor ligands determines the functional relevance of TRAIL and TNFα signaling for IFNα/BV6-induced cell death. Second, we demonstrate cooperative induction of cell death by IFNα/BV6 cotreatment in several solid cancers such as glioblastoma, colon and pancreatic carcinoma, rhabdomyosarcoma and Ewing sarcoma. Together with our previous report showing synergistic antileukemic activity of IFNα and BV6 in AML, a hematological malignancy [9], our present study provides evidence showing that this dual immunotherapy approach is of general relevance across cancers.

Recently, there has been a renewed interest in therapeutically exploiting type I interferons such as IFNα, which is the first bio-therapeutic approved and that plays a documented role as anticancer agent in solid cancers as well as in hematological malignancies [6]. Modified IFNα preparations that yield constant IFN levels over a prolonged period of time exhibited substantial anticancer activity in preclinical *in vivo* models as well as in a proof-of-concept study in humans [12, 13].

Besides IFNs, innate immune stimuli including oncolytic viruses and immunostimulatory adjuvants have been used in combination with Smac mimetics for cancer immunotherapy. Recently, oncolytic virus infection or treatment with non-infectious adjuvants such as poly(I:C) and CpG oligonucleotides have been reported to engage bystander cancer cell death upon concomitant treatment with Smac mimetics via stimulation of a cytokine storm [14]. Furthermore, Bacillus Calmette-Guérin (BCG)-based immunotherapy has been described to act in concert with Smac mimetics to suppress tumor growth of bladder cancer [15]. These reports underline the therapeutic potential of Smac mimetic-based combinations with immune stimuli for cancer immunotherapy.

Our study has important implications for the development of dual cancer immunotherapy with Smac mimetics. First, by demonstrating a synergistic interaction of IFNα/Smac mimetic combination therapy in a variety of solid cancers, our findings imply that this approach is generally relevant to maximize the anticancer activity of Smac mimetics. Second, IFNα/Smac mimetic cotreatment could offer a treatment option that may be safer than Smac mimetic monotherapy, since it relies on autocrine/paracrine TNFα production or TRAIL signaling. Since TNFα not only mediates Smac mimetic-induced cell death but may also cause dose-limiting, on-target side effects such as cytokine release syndrome [16], TNFα production represents a double-edged sword. Due to synergistic drug interactions, IFNα/Smac mimetic combination therapy allows to use lower doses of each individual agent, which might reduce the risk of dose-dependent toxic side effects. In addition, IFNα/Smac mimetic combination may alter the spectrum of treatment-induced cytokine generation and could shift the production towards TRAIL, a well-established IFN-stimulated gene (ISG) downstream of type I IFN signaling [17, 18]. Also, we recently identified Smac mimetic-stimulated, NF-κB-dependent upregulation of DR5 as a critical mediator of Smac mimetic-induced apoptosis, for example in A172 cells [19]. Thus, dual immunotherapy using Smac mimetics and IFNα may provide a mean to engage the TRAIL signaling pathway to cell death endogenously in cancer cells. Of note, combinations of Smac mimetics together with the administration of exogenous TRAIL receptor agonists were shown in a variety of preclinical cancer models to efficiently suppress tumor growth [20]. Currently, the Smac mimetic Birinapant and the TRAIL-R2 agonist Conatumumab are under clinical evaluation [21]. Since in clinical trials TRAIL receptor agonists proved to be well-tolerated in humans [22], Smac mimetic-based combination regimens that embark on TRAIL signaling to mediate cell death may offer a favorable safety profile.

Third, our findings are relevant for the development of suitable biomarkers for clinical trials with Smac mimetics. Upregulation of circulating cytokines such as TNFα in the blood of patients receiving Smac mimetics has been used as an indicator of pharmacodynamic activity of Smac mimetics, reflecting on-target engagement of signaling pathways [16, 23]. Our study implies that determination of TNFα production may not be sufficient and that TRAIL production and sensitivity has to be taken into consideration as a biomarker for IFNα/Smac mimetic cotreatment, too. Noteworthy, sensitivity towards TRAIL ligand has recently been implicated as a marker for responsiveness to TRAIL/Smac mimetic combination treatment in breast carcinoma [24]. In conclusion, our study provides novel insights on how immunostimulatory drugs such as IFNα might enhance the efficacy of Smac mimetics which might support the use of these drugs in the treatment of cancer.

## MATERIALS AND METHODS

### Cell culture and chemicals

Human cell lines were obtained from American Type Culture Collection (ATCC;CEM, Manassas, VA, USA) or Deutsche Sammlung von Mikroorganismen und Zellkulturen (DSMZ; Braunschweig, Germany) and cultured in DMEM medium or RPMI 1640 (Life Technologies, Inc., Eggenstein, Germany) or McCoy's medium (Invitrogen, Karlsruhe, Germany) supplemented with 10% fetal calf serum (FCS) (Biochrom, Berlin, Germany), 1 mM Sodium Pyruvate (Invitrogen, Karlsruhe, Germany) and 1% penicillin/streptomycin (Invitrogen, Karlsruhe, Germany). IFNα was obtained from Sigma-Aldrich (Taufkirchen, Germany), zVAD.fmk from Bachem (Heidelberg, Germany), Nec-1 from Merck (Darmstadt, Germany) and Enbrel from Pfizer (Berlin, Germany). The bivalent Smac mimetic BV6 was kindly provided by Genentech, Inc. (South San Francisco, CA, USA). All other chemicals were obtained from Sigma-Aldrich or Carl Roth (Karlsruhe, Germany) unless indicated otherwise.

### Western blot analysis and immunoprecipitation

Western blot analysis was performed as described previously [25] using the following antibodies: mouse anti-XIAP, mouse anti-RIP1, mouse anti-FADD from BD Biosciences (Heidelberg, Germany), goat anti-cIAP1 from R&D Systems (Wiesbaden, Germany), rat anti-cIAP2, mouse anti-caspase 8 from Enzo Life Sciences (Lörrach, Germany), rabbit anti-caspase-3 from Cell Signaling Technology (Danvers, USA), rabbit anti-caspase 8 from Epitomics (Burlingame, USA) and mouse anti-GAPDH from HyTest (Turku, Finnland) followed by goat-anti-mouse IgG or goat-anti-rabbit IgG conjugated to horseradish peroxidase purchased from Santa Cruz Biotechnology, Inc. (Dallas, USA). Enhanced chemiluminescence was used for detection from Amersham Bioscience (Freiburg, Germany). Further, donkey anti-mouse IgG, donkey anti-rabbit IgG or donkey anti-goat IgG labeled with IRDye infrared dyes were used for fluorescence detection at 700 nm 800 nm (LI-COR Biotechnology, Bad Homburg, Germany). Immunoprecipitation was performed as previously described [26] using RIPA buffer or NP-40 buffer for cell lysis.

### Determination of cell death and cell viability

Cell death was determined by the analysis of DNA fragmentation of PI-stained nuclei or by PI staining to determine plasma membrane permeabilization using flow cytometry (FACSCanto II, BD Biosciences) as described previously measuring a total of 10^4^ events per sample [27]. Cell viability was measured by 3-(4,5-dimethylthiazol-2-yl)-2,5-diphenyltetrazolium bromide (MTT) assay according to the manufacturer's instructions (Roche Diagnostics, Mannheim, Germany).

### Determination of mRNA levels and protein levels of TNFα and TRAIL

TNFα and TRAIL mRNA levels were determined by quantitative real-time PCR (qRT-PCR) analysis. Total RNA extraction and cDNA synthesis were performed as previously described [28]. TNFα and TRAIL mRNA levels were assessed by Taqman Gene Expression Assay purchased from Life Technologies (TNFα: Hs01113624_g1, TRAIL: Hs00921974_m1) and the levels of 28S rRNA by SYBR®Green qPCR assay from Applied Biosystems (Darmstadt, Germany) according to the manufacturer's instructions using the 7900HT fast real-time PCR system from Applied Biosystems (Darmstadt, Germany); 28S rRNA forward primer: TTGAAAATCCGGGGGAGAG; reverse primer: ACATTGTTCCAACATGCCAG. The relative expression of the target gene transcript and reference gene transcript was calculated as ΔΔC_t._ 28S rRNA was used as reference gene. TRAIL protein levels in whole cell lysates were determined by Human TRAIL/TNFSF10 Quantikine ELISA Kit obtained from R&D Systems (Wiesbaden, Germany) and TNFα levels in the supernatant of cells were determined by Human TNFα Ultrasensitive ELISA Kit purchased from Invitrogen (Carlsbad, USA) according to the manufacturer's instructions.

### Flow cytometric analysis of cell surface expression of DR5

Fluorescence-activated cell sorting (FACS) analysis of cell surface expression of DR5 was done using PE-conjugated DR5 antibody (R&D Systems). In brief, cells were harvested, washed with PBS and incubated in 25 μl wash buffer (PBS containing 1% goat serum) for 5 min at room temperature. Afterwards, cells were incubated with 10 μg/ml anti-DR5 antibody or mouse IgG1-PE as isotype control for 30 min at 4°C in the dark. Cells were then washed with wash buffer and resuspended in 5 μl/ml biotinylated F(ab)2 fragments diluted in wash buffer. After 30 min incubation at 4°C, cells were washed with wash buffer and incubated in streptavidin-PE for 30 min at 4°C in the dark. Cells were washed in wash buffer and resuspended in 100 μl PBS for final analysis by flow cytometry.

### Gene silencing

For transient knockdown by siRNA, cells were reversely transfected with 5 nM SilencerSelect siRNA (Invitrogen) control siRNA (# 4390843) or targeting siRNAs (s16663 and s16665 for TRAIL, s16651 and s16653 for RIP1, s16756 and s16758 for DR5) using Lipofectamine RNAi Max from Invitrogen (Carlsbad, USA) and OptiMEM from Life Technologies (Darmstadt, Germany).

### Statistical analysis

Statistical significance was assessed by Student's t-test (two-tailed distribution, two-sample, unequal variance). Drug interactions were analyzed by the CI method based on that described by Chou [29] using CalcuSyn software (Biosoft, Cambridge, UK). CI <0.9 indicates synergism, 0.9-1.1 additivity and >1.1 antagonism. IC50 values were calculated by Origin data analysis software (OriginLab, Northampton, MA, USA).

## SUPPLEMENTARY MATERIAL FIGURES AND TABLE


