# Supplementary material for: Cooperative TRAIL production mediates IFNα/Smac mimetic-induced cell death in TNFα-resistant solid cancer cells
Source: Oncotarget. 2016 Jan 13;7(4):3709–25. doi: 10.18632/oncotarget.6915 (PMC4826164; doi:10.18632/oncotarget.6915)
Supplement: Supplementary file 1 [file oncotarget-07-3709-s001.pdf]

Cooperative TRAIL production mediates IFNα/Smac mimetic-induced cell death in TNFα-resistant solid cancer cells

Supplementary Material

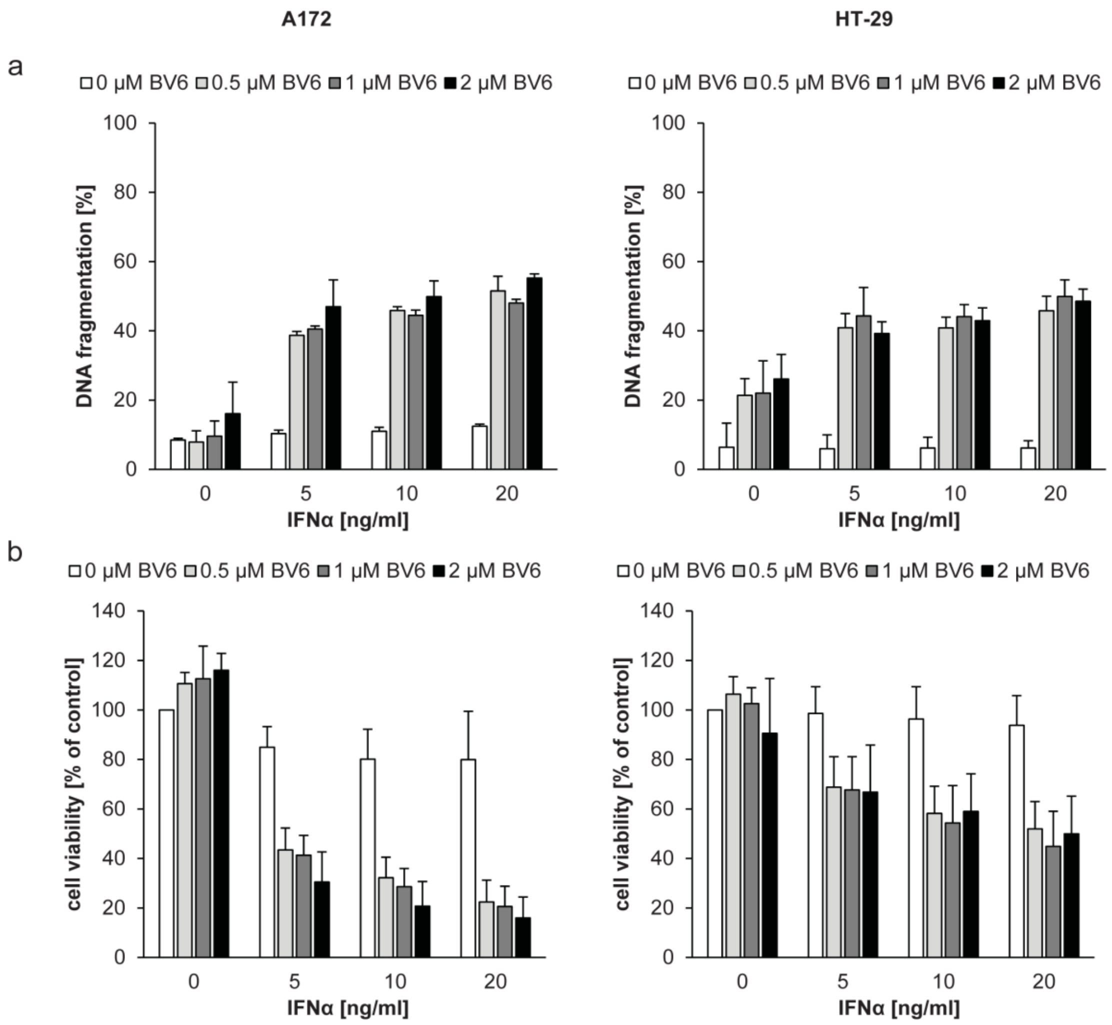

Suppl. Fig. 1. IFNα/BV6 cotreatment synergistically induces cell death in A172 and HT-29 cells.
A172 and HT-29 cells were treated for 72 hours with indicated concentrations of IFNα and/or BV6. Cell death was determined by analysis of DNA fragmentation using flow cytometry and mean + SD of three independent experiments performed in duplicate are shown (A). Cell viability was determined by MTT assay and data are shown as percentage of untreated control cells with mean + SD of three independent experiments performed in triplicate (B).

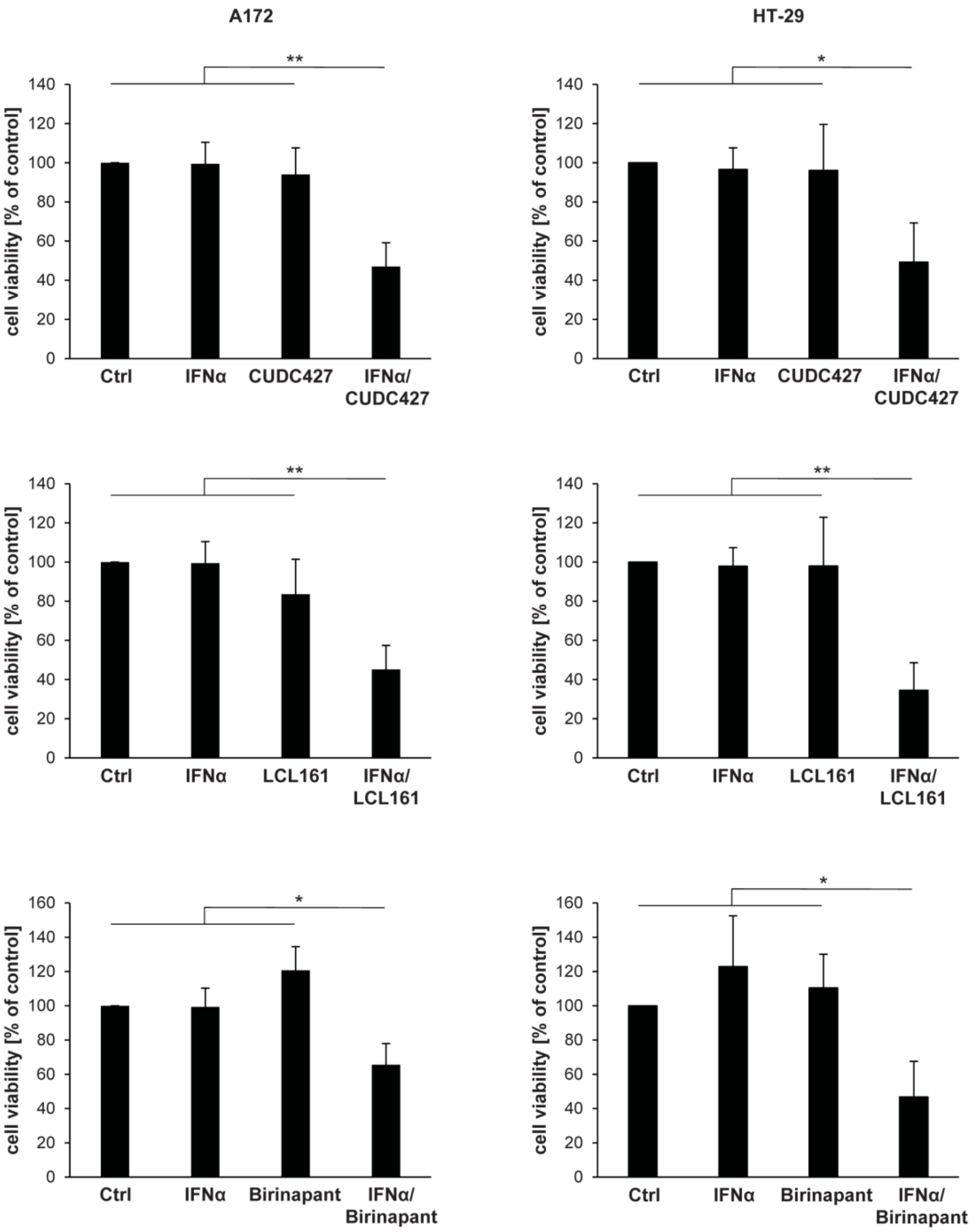

Suppl. Fig. 2. Different Smac mimetics cooperate with IFNα to reduce cell viability.
A172 and HT-29 cells were treated with 10 ng/ml IFNα and/or CUDC427, LCL161 (A172: 30 µM; HT-29: 10 µM) or 5 µM Birinapant for 72 hours. Cell viability was determined by MTT assay and data are shown as percentage of untreated cells with mean + SD of three independent experiment performed in triplicate; * $P < 0.05$; ** $P < 0.01$.

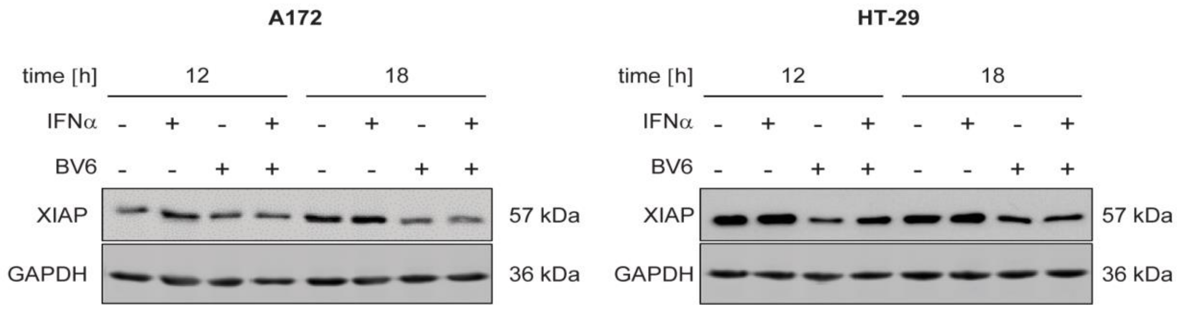

Suppl. Fig. 3. Effect of IFNα and/or BV6 on XIAP protein expression.
Cells were treated for 12 and 18 hours with IFNα (A172: 5 ng/ml, HT-29: 10 ng/ml) and/or 1 μM BV6. Protein levels of XIAP were assessed by Western blotting, GAPDH was used as loading control.

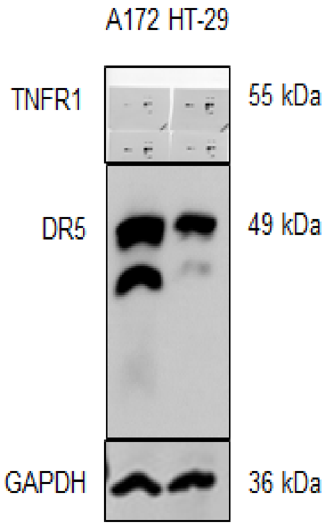

Suppl. Fig. 4. TNFR1 and DR5 expression.
Constitutive protein levels of TNFR1 and DR5 were assessed in untreated cells by Western blotting, GAPDH was used as loading control.

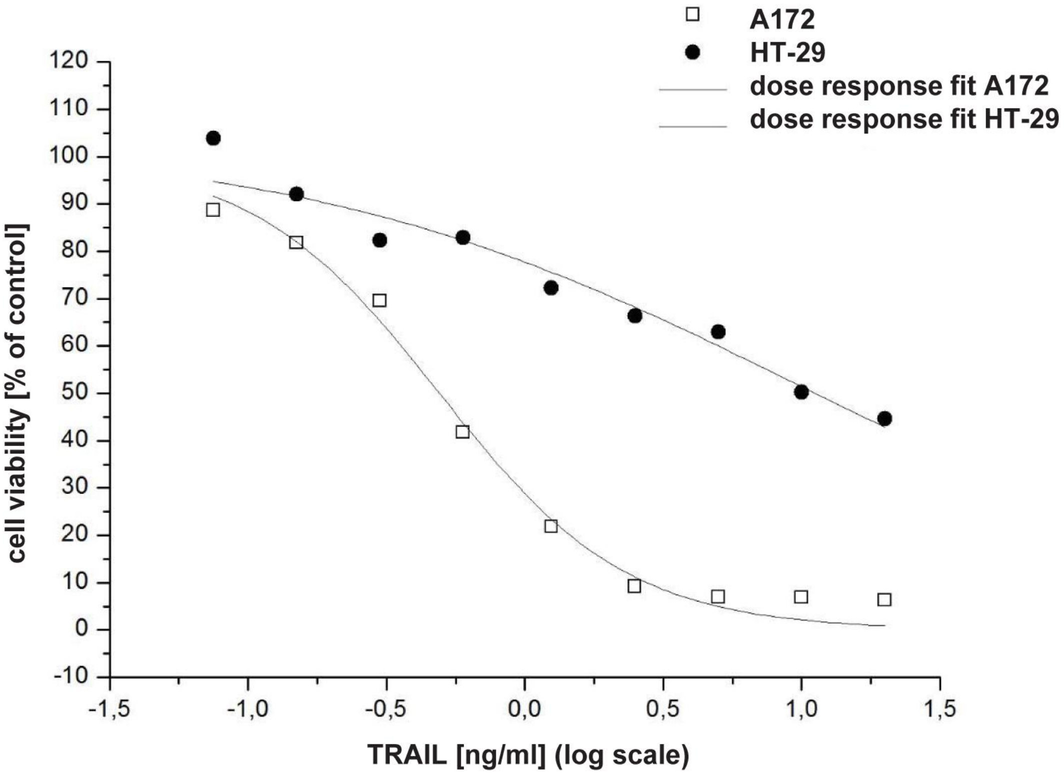

Suppl. Fig. 5. Calculation of IC50 values for TRAIL/BV6 cotreatment.
IC50 values for TRAIL/BV6 cotreatment were calculated by Origin data analysis software (OriginLab, Northampton, MA, USA).

Suppl. Tab. 1. Synergistic induction of cell death by IFNα and BV6.
Combination indices (CI) were calculated as described in Materials and Methods for DNA fragmentation (A) and cell viability (B) shown in suppl. Fig. 1 after treatment with indicated concentrations of IFNα and BV6. CI <0.9 indicates synergism, 0.9-1.1 additivity and >1.1 antagonism.

a

A172

		BV6 [µM]		
		0.5	1	2
IFNα [ng/ml]	5	0.04	0.007	0.011
	10	0.002	0.005	0.007
	20	0.001	0.003	0.003

HT-29

		BV6 [µM]		
		0.5	1	2
IFNα [ng/ml]	5	0.006	0.006	0.034
	10	0.006	0.006	0.015
	20	0.002	0.002	0.005

b

A172

		BV6 [µM]		
		0.5	1	2
IFNα [ng/ml]	5	0.064	0.04	0.056
	10	0.042	0.075	0.072
	20	0.027	0.051	0.076

HT-29

		BV6 [µM]		
		0.5	1	2
IFNα [ng/ml]	5	0.067	0.026	0.044
	10	0.068	0.022	0.039
	20	0.021	0.023	0.038
